# The role of the circadian clock in cancer hallmark acquisition and immune-based cancer therapeutics

**DOI:** 10.1186/s13046-021-01919-5

**Published:** 2021-04-01

**Authors:** Elizabeth Cash, Sandra Sephton, Cassandra Woolley, Attia M. Elbehi, Anu R. I., Bene Ekine-Afolabi, Victor C. Kok

**Affiliations:** 1 Department of Otolaryngology and Communicative Disorders, University of Louisville School of Medicine, James Graham Brown Cancer Center, 529 S Jackson Street, Louisville, KY 40202 USA; 2 Department of Psychological & Brain Sciences, University of Louisville, Louisville, KY USA; 3 Department of Microbiology and Immunology, University of Louisville School of Medicine, Louisville, KY USA; 4 Department of Oncology, Medical Sciences Division, University of Oxford, Oxford, UK; 5 Department of Clinical Biochemistry, MVR Cancer Center and Research Institute, Kerala, India; 6 ZEAB Therapeutic Ltd, London, UK; 7 Department of Health, Sport & Bioscience, University of East London, Stratford, UK; 8 Department of Medical Oncology, Kuang Tien General Hospital Cancer Center, Taichung, Taiwan; 9 Department of Bioinformatics and Medical Engineering, Asia University Taiwan, Taichung, Taiwan

**Keywords:** Circadian, Cancer, Immuno-oncology, Immune checkpoint inhibitor, Glucocorticoid, Clock gene

## Abstract

The circadian system temporally regulates physiology to maintain homeostasis. Co-opting and disrupting circadian signals appear to be distinct attributes that are functionally important for the development of a tumor and can enable or give rise to the hallmarks that tumors use to facilitate their initiation, growth and progression. Because circadian signals are also strong regulators of immune cell proliferation, trafficking and exhaustion states, they play a role in how tumors respond to immune-based cancer therapeutics. While immuno-oncology has heralded a paradigm shift in cancer therapeutics, greater accuracy is needed to increase our capability of predicting who will respond favorably to, or who is likely to experience the troubling adverse effects of, immunotherapy. Insights into circadian signals may further refine our understanding of biological determinants of response and help answer the fundamental question of whether certain perturbations in circadian signals interfere with the activity of immune checkpoint inhibitors. Here we review the body of literature highlighting circadian disruption as a cancer promoter and synthesize the burgeoning evidence suggesting circadian signals play a role in how tumors respond to immune-based anti-cancer therapeutics. The goal is to develop a framework to advance our understanding of the relationships between circadian markers, cancer biology, and immunotherapeutics. Bolstered by this new understanding, these relationships may then be pursued in future clinical studies to improve our ability to predict which patients will respond favorably to, and avoid the adverse effects of, traditional and immune-based cancer therapeutics.

## Introduction to circadian signals in Cancer

Mammalian physiology fluctuates with the earth’s rotation, allowing species to capitalize on the availability of nutritive and environmental resources. Daily, or circadian, fluctuations are evident in all mammalian somatic cells. The body’s central circadian clock, located in the suprachiasmatic nucleus (SCN) of the hypothalamus, is the primary circadian synchronizer [1]. Environmental light stimulates specialized retinal ganglion cells that signal the SCN to send circadian reset signals (via hormones) to the periphery. These signals coordinate physiological homeostasis and adaptation to both external and internal fluctuations. While some intracellular circadian rhythms can be self-sustaining, SCN reset signals combined with other hormonal and metabolic cues are responsible for cellular synchrony with the natural environment [2].

Circadian cycling is evident in all somatic cells. While there are a substantial number of cancer-relevant genes whose expression cycles in a circadian fashion, here we focus on set of core clock genes known to control cell cycle timing (Table 1 [51];. Clock genes regulate the cell cycle by controlling expression of cell cycle regulators and checkpoint controllers, including p21, WEE1, p16/INK4a, CDK2, CDK4, Cyclin D1, p27, and p57 [52]. Clock genes are aptly named as they undergo regulated, daily cycles of activation and expression. These daily cycles are thought to be crucial for cell cycle entry/exit, control, and to protect DNA from hazards during susceptible periods in the cell cycle [53, 54]. Cellular mitosis occurs in specific, circadian time windows, further suggesting tight coupling between circadian cycles and cell division [52].

**Table 1:** Core circadian clock markers and their demonstrated links to cancer hallmarks and immune function

Name	Description	Experimental data highlighting how each circadian signal ties to multiple cancer hallmarks*
**Core circadian clock genes**		
BMAL1/2 (ARNTL)	Positive regulator of circadian cycles	1. Downregulation [3] or mutation [4] upregulates MYC in vivo, knockout increases cellular senescence in vivo [5]
		2. Knockdown upregulates cyclin D1 expression in vitro [6], downregulation accelerates cell cycling in vitro [7]
		3. Downregulation decreases apoptosis in vitro [7], knockout permits uncontrolled Atg14-mediated initiation of autophagy in vivo [8]
		4. Knockout causes SIRT1-mediated telomere shortening in vivo [9]
		6. Downregulation promotes metastatic (i.e., rapidly proliferating) phenotype in vitro [7]
		7. Downregulation permits upregulation of WEE1 and TP53 in vivo [3]
		8. Knockdown reduces tumor NAD+ levels in vitro [10]
		9. Knockdown induces expression of pro-inflammatory angiopoietin-like protein 2 in vivo [11], knockout permits uncontrolled proinflammatory T_H_17 cell development via IL-21 in vivo [12], and alters T_H_17 cell differentiation via RORγt and NFIL3 pathways in vivo [13]
CLOCK	Positive regulator of circadian cycles	3. Knockout permits uncontrolled Atg14-mediated initiation of autophagy in vivo [8]
		4. Knockdown reduces tumor NAD+ levels in vitro [10]
		7. Knockout deregulates WEE1 transcription in vivo [14]
		9. Knockout permits uncontrolled differentiation of T_H_17 cells via RORγt and NFIL3 pathways [13]
		10. Knockout reduces T_H_1 cell counts in vivo [13]
PER1/2/3 (period)	Repressor of circadian cycles	1. Knockout increases RAS expression in vivo [15], overexpression downregulates PI3K in vivo [16]
		2. Overexpression inhibits tumor growth in vivo [16], downregulation causes overexpression of MYC in vivo [17], knockdown increases multiple cyclins in vitro [18, 19]
		3. Knockout downregulates P53-mediated apoptosis in vivo [20]
		4. Overexpression increases β-catenin in vivo [16]
		5. Knockdown increases VEGF in vitro [21]
		6. Downregulation activates EMT [22], TWIST1/2, SLUG, and ZEB1/2 in vitro [23]
		7. Downregulation upregulates P53 in vivo and in vitro [17, 23], and upregulates WEE1 in vivo [3], while knockout deregulates rhythmic expression of WEE1 in vivo [15]
		8. Downregulation reprograms metabolism (downregulates glycolysis and lactate excretion) in vivo [24]
		9. Downregulation activates MMP1 in vitro [23], knockout increases IL-6 and TNF-α in vivo [15]
		10. Downregulation increases immunosuppressive T_REG_ in primary in vivo tumors [25]
CRY1/2 (cryptochrome)	Repressor of circadian cycles	2. Knockdown represses cyclin D1 expression [6], permits Rb phosphorylation [6], and inhibits ubiquitination and turnover of c-Myc in vitro [26]; mutation downregulates c-Myc in vivo [4]
		3. Knockdown alters expression of BCL2 in vitro [27], knockout permits uncontrolled Atg14-mediated initiation of autophagy in vivo [8]
		7. Knockout deregulates WEE1 transcription in vivo [14] knockdown leads to the accumulation of DNA damage [27] and alters p53 and p21 expression and transcription in vitro [28]; knockout elevates proinflammatory cytokines in vitro [29]
		10. Downregulation increases immunosuppressive T_REG_ in primary in vivo tumors [25]
**Circadian Receptors**		
RORA/B/C (retinoic acid receptor-related orphan receptor α/β/γ; NR1F1/2/3)	Enhances rhythmic expression of BMAL1 and BMAL2	7. Downregulation decreases P53 expression in vitro [30]
		8. Mutation permits loss of HDAC3 co-repression of metabolism genes [31]
		9. Knockdown impairs IL-17 expression and T_H_17 cell development in vivo and in vitro [32], RORγ agonist activates T_H_17 cells and attenuates immunosuppression in vitro [33]
REV-ERBA/B (NR1D1/2)	Represses rhythmic expression of BMAL1 and BMAL2	2. Agonist suppresses cyclin A expression in vitro [34]
		3. Agonist inhibits autophagy in vitro [35]
		4. Agonist reduces apoptosis in vitro [36]
		6. Downregulation increases cell proliferation, motility and micro-metastasis formation in vivo [3]
		9. Knockdown impairs IL-17 expression and T_H_17 cell development in vivo and in vitro [32], knockout alters T_H_17 cell differentiation via RORγt and NFIL3 pathways in vivo [13], knockdown or agonist gate expression and release of IL-6 in vivo and in vitro [37]
**Circadian Hormones**		
Glucocorticoids	Positive regulator of diurnal behaviors (e.g., activity); immunosuppressive	1. Reintroducing rhythmic expression decreases S-phase cycling in vitro [38], dysregulation induces epidermal growth factor receptor (EGFR) overexpression in vivo [39]
		2. Dysregulation induces G1/S cell cycle progression markers MYC, CDK3, CCND3, CCND1 and CDT1; upregulates Rb expression, phosphorylation in vitro [6]
		5. Dexamethasone inhibits tumor cell VEGF and IL-8 expression in vivo [40], stress-induced overexpression induces angiogenesis in vivo [41]
		6. Overexpression induces metastatic colonization in vivo [42]
		7. Stress-induced overexpression induces nitric oxide-mediated DNA damage in vivo [41]
		8. High-dose dexamethasone decreases expression of glucose uptake and glycolysis genes in vivo [43]
		10. High-dose dexamethasone decreases expression of anti-tumor immune response genes in vivo [43], over-expression by tumor cells suppresses immune cell function in vitro [44], stress-induced overexpression induces pro-tumorigenic M2 macrophage upregulation in vivo [41]
Melatonin	Positive regulator of nocturnal behaviors (e.g., sleep)	1. Loss of expression permits greater EGFR/MAPK pathway activity in vivo [45]
		3. Exposure reduces AMPK and autophagic activity in vitro [46]
		4. Loss of expression permits cytotoxicity and apoptosis in vivo [45]
		7. Suppression increases LINE-1 retrotransposon-induced DNA damage in vitro [47]
		8. Dysregulation accelerates tumor metabolism, increases aerobic glycolysis in vivo [48]
		9. Administration selectively activates T_H_1 (IL-2 and IL-6 in lymphocytes and monocytes), but not T_H_2, cells in vitro [49], and T_H_17 differentiation via NFIL3 pathway [50]

Links to cancer hallmarks are reported by number: 1, Sustained proliferative signaling; 2, Evading growth suppressors; 3, Resisting cell death; 4, Enabling replicative immortality; 5, Inducing/sustaining angiogenesis; 6, Activating invasion/metastasis; 7, Genome instability/mutation; 8, Deregulating cellular energetics; 9, Tumor-promoting inflammation; 10, Avoiding immune destruction

When cue mismatches occur (e.g., chronic exposure to light at night), they can lead to widespread dysregulation of physiological cycling. Indeed, circadian signals are disrupted across different tumor types [55], typically via DNA hypermethylation [56], histone modification, or changes in chromatin conformation and interactions [57]. Such circadian disturbances allow tumor cells to cycle at variable rates. Given that cancer is often thought of as a disease of impaired cell cycle exit, disruptions to circadian signals likely compound cell cycle exit impairments to accelerate cell cycle progression, thereby permitting more rapid tumor initiation, growth and spread. Disrupted circadian signaling has been implicated in the acquisition or progression of multiple cancer hallmarks [58], including: 1) sustained proliferative signaling, 2) evading growth suppressors, 3) resisting cell death, 4) enabling replicative immortality, 5) inducing/sustaining angiogenesis, 6) activating invasion/metastasis, 7) genome instability/mutation, 8) deregulating cellular energetics, 9) tumor-promoting inflammation, and 10) avoiding immune destruction. A summary of primary, experimental literature supporting these relationships is presented in Table 1.

The extensive literature reveals strong and consistent results across human studies and murine models, where clinical observations are coherent, plausible, and backed by experimental evidence. Chronic and subtle – but perhaps not acute – circadian disruption increases cancer incidence in humans and mice, suggesting a potential biological gradient. Effects are reversible, though some converse and bidirectional signaling have also been observed [54]. While the sequelae of disrupted circadian signaling are generally analogous across human cancer types, experimental evidence of context-specific effects suggests further investigation is needed. For example, some cancer types may require regulated circadian signals in order to develop [59], suggesting that tumors may actually be reprogramming and using circadian signals rather than simply disrupting or downregulating them [54, 55]. Whether circadian disruption occurs in advance of, or in tandem with, other hallmark processes has also not been clearly established, complicating determinations of specificity and temporal precedence. However, there is sufficient evidence to support the notion that disrupted or reprogrammed circadian signaling is not simply a byproduct of malignancy, but an enabling characteristic that tumors use to facilitate their initiation, growth and progression. Improvements in the clinical measurement of circadian disruption, in tandem with ongoing advances cancer epigenomics and metabolomics, will lead to a greater understanding of these nuanced relationships.

More specifically, there is extensive evidence linking circadian disruption to the hallmarks of inflammation and immune evasion (also highlighted in Table 1). Circadian genetic and hormonal signals strongly influence - and indeed control - a broad variety of systemic immune function, including activation of innate and adaptive immunity; immune cell migration, trafficking, differentiation, receptor interactions, and signaling; cytotoxicity; and inhibitory/regulatory function [2, 60]. At the level of the tumor, circadian clock genes are strongly related to immune signaling, activation, and immunophenotype across multiple cancer types [56]. Circadian hormones also appear to simultaneously influence systemic and local tumor functions [61], highlighting the connections between circadian-immune signaling relationships in both healthy and malignant cellular environments. It therefore stands to reason that circadian signals play a role in how tumors respond to immune-based cancer therapeutics.

## Circadian disruption: specific consequences for immunotherapy

Immunotherapies boost host anti-tumor immunity and have heralded a revolution in anti-cancer strategies. However, we lack the ability to consistently determine which patients will respond favorably to, or who is likely to experience the troubling adverse effects of, immunotherapy. Greater accuracy therefore is needed to increase our capability of predicting these responses.

**Fig. 1:**
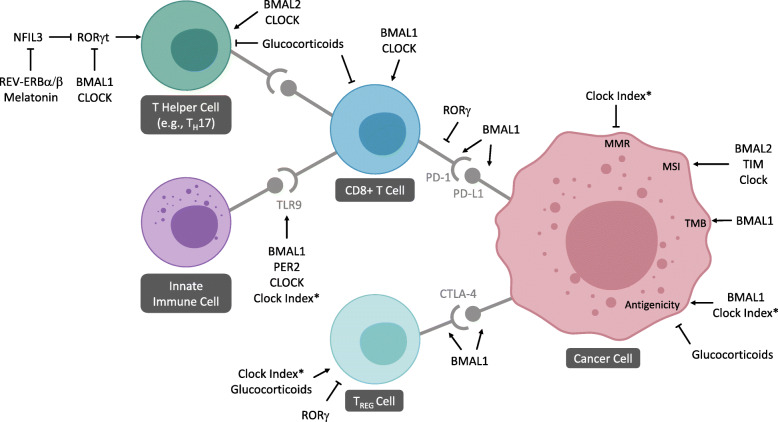
Circadian markers and their influences on immune-oncology pathways. Diagram represents a simplified depiction highlighting immune cells and receptors of interest. Circadian effects supported by experimental evidence are depicted, with connectors representing the direction of relationships (i.e., arrows represent positive or upregulatory relationships, flat connectors represent negative or inhibitory relationships). Not depicted, disruption or tumor reprogramming of circadian gene signals has been associated with tumor immunophenotype patterns and glucocorticoids have been implicated in poorer response to anti-PD-(L)1 therapy. *“Clock Index” refers to a combined index score of BMAL1, PER1/2/3, CRY1/2, and CLOCK expression levels across multiple cancer types [56]

Not surprisingly, the relationships between circadian rhythm, tumor growth, and immune function may have major implications for anti-cancer immunotherapy outcomes. Insights into circadian signals may further refine our understanding of biological determinants of response and help explain whether certain perturbations in circadian signals interfere with the activity of immune checkpoint inhibitors. The fundamental question of how this coupling may potentially affect heterogeneous responses to cancer immunotherapeutics remains to be addressed. In the following sections, we synthesize the emerging evidence suggesting circadian signals play a role in how tumors respond to immune-based anti-cancer therapeutics, with the goal of developing a framework to advance our understanding of the relationships between circadian markers and immunotherapeutics. Relationships reviewed are highlighted in Fig. 1.

## Circadian signals and genetic determinants of tumor immunogenicity

Owing to their myriad mutations and alterations, cancer cells have several characteristics that cause them to become increasingly antigenic [62]. DNA mismatch repair (MMR) may become error-prone, resulting in microsatellites, short motifs of up to 6 DNA nucleotides that form loops when DNA strands are rejoined. This microsatellite instability (MSI) accumulates; often, resulting in higher tumor mutational burden (TMB), greater neoantigen expression, and greater CD8+ lymphocyte infiltration. In general, cancers with the highest TMB are the most immunotherapy responsive and are thought to be good candidates for immune checkpoint inhibition [63].

Circadian signaling has been linked to each of the tumor characteristics of high MSI, MMR deficiency, and high TMB. In colorectal tumors, BMAL2 upregulation was associated with higher levels of the neoplastic growth promoter SERPINE1, which promotes MSI [64]. This is consistent with experimental evidence suggesting that upregulated BMAL2 indeed drives promoters of MSI [65]. Analysis of multiple human cancer (TCGA) samples revealed that an index of higher core circadian clock gene (BMAL1, PER1/2/3, CRY1/2, CLOCK) expression is related to downregulated MMR pathways, possibly indicating the occurrence of fewer mismatch events, though this was not directly assessed [56]. TMB was adjusted in this analysis to reduce variation present across the cancer types assessed, and thus was also not directly addressed. However, other studies link high TMB to circadian signaling. For example, higher BMAL1 expression is positively correlated with greater TMB in metastatic melanoma [66].

Immunotherapy often works best for patients who have demonstrated a pre-existing anti-tumor immune response. Circadian signaling may influence the recognition of tumor cells for this anti-tumor response. In multiple cancer types, core circadian clock gene expression has been linked to the pathways of tumor antigen processing/presentation, tumor immunogenicity, and HLA phenotype; though whether these pathways are upregulated or downregulated depends on tumor type [56]. For example, though circadian gene expression is significantly downregulated across malignant cells in general, overexpression of circadian genes was observed in head and neck carcinoma, lung adenocarcinoma, renal clear cell carcinoma, and kidney chromophobe cancers [56]. Similarly, higher BMAL1 expression is positively correlated with greater TMB and increased antigen presentation, but not MMR pathway activation in metastatic melanoma [66]. Regarding circadian humoral effects, murine models reveal that social stress induces circulating glucocorticoids to cause downregulation of genes involved in tumor antigen presentation [67]. Whether circadian hormones influence MMR, MSI, or TMB remains to be determined.

Clinical data lend some functional validation to the studies on genetic determinants. Metastatic melanoma patients with high BMAL1 expressing tumors demonstrate improved survival response to anti-PD-1 immunotherapy compared to low BMAL1 expressing patients (50% overall survival at ~ 105 months vs ~ 70 months, respectively; 66). Expression of the circadian gene TIM (Timeless) is directly and positively associated with the presence of MSI in colorectal tumors [68]. CLOCK is mutated or downregulated in about half of MSI-high colorectal tumors. Amplification of TIM and attenuation of CLOCK have both been shown to attenuate DNA repair triggered by therapeutically induced breaks [68, 69]. As objective response rates to immunotherapy for MSI-high tumors have been reported to be around 50% [70], differential expression levels of TIM and CLOCK genes could potentially explain variance in responses observed among MSI-high patients. In future work, response rates among tumors with high BMAL2, TIM, or CLOCK expression levels could be compared to those with low BMAL2, TIM, or CLOCK levels, and then cross-tabulated against MSI level to determine the predictive capacity of circadian gene expression fluctuations. If this signal is further validated, a multifactorial biomarker panel that combines assessment of these circadian markers in the context of MSI level may provide stronger predictive value for therapeutic response than using MSI as marker alone.

Together these data suggest that circadian gene and hormone expression is closely associated with the manifestation of tumor immunogenicity and may interact with treatments that target it, including chemotherapeutic and immunotherapeutic agents. Continued experimental validation of potential distinct biological processes driving these changes may enable causal determinations to be made.

## Circadian clock genes and immunotherapies

Tumors escape the host immune system by downregulating certain immune capabilities to induce tolerance. Tumors express ligands, such as PD-L1, on their surface to inhibit inflammatory responses from infiltrating lymphocytes. PD-L1 binds to the lymphocyte PD-1 receptor and induces a state of exhaustion. While healthy tissue demonstrates time-dependent (i.e., circadian) expression of PD-L1 [71], tumors can either constitutively express PD-L1 on all of their cells in an innately immune-resistant fashion, or they can be adaptively induced to express PD-L1 by adjacent lymphocytes [62]. Across multiple cancer types, greater circadian clock gene expression is associated with variation in the levels of immune checkpoint and effector cells [56]. In a pan-cancer analysis, a broad variety of cancer types were shown to downregulate circadian gene expression, causing a direct regulatory effect on PD-L1 expression and perpetuating an immunosuppressive tumor phenotype [72]. The nature and direction of these relationships varies by cancer type, suggesting these influences are dependent on both the disease and the context.

In melanoma, higher BMAL1 is associated with higher T-lymphocyte infiltration, higher PD-1 and PD-L1 expression, better objective response to anti-PD1 immunotherapy and improved overall patient survival [66]. In thoracic cancers, circadian genes BMAL1 and CLOCK correlate with the infiltration level of CD8+ T-cells [71]. Circadian pharmacologic studies show that RORγ agonists, which can activate BMAL1 transcription [73], attenuate the expression of PD-1 receptors [33]. The RORγ agonist LYC-55716 has demonstrated initial success in Phase 1 clinical trials as a single agent for locally advanced or metastatic solid tumors of multiple types (clinical trial NCT02929862): good tolerability, safety, and pharmacokinetics were observed, as well as partial responses in 6% patients and disease stabilizations in 34% patients over 12-month follow-up [74]. A similar Phase 1 trial of LYC-55716 used in combination with pembrolizumab for non-small cell lung cancer is underway (NCT03396497). These data suggest tight but complex circadian control over immune checkpoint signaling in the tumor microenvironment and highlight BMAL1 expression as a predictive biomarker as well as a promising enhancer of anti-PD-1 immunotherapy. Through their effects on PD-1 receptor expression and increased anti-tumor immunity via upregulated cytokines, effector and memory cells, pharmacologic manipulation of circadian signals may represent a promising area for enhancement of immunotherapy response.

Toll like receptor (TLR) agonists are being developed for use as adjuvants to checkpoint inhibition and have started to enter the clinic. Intratumoral injection of TLR9 enhances response to PD-L1 blockade in metastatic head and neck tumors in mice [75] as well as in a Phase 1b clinical trial for metastatic melanoma [76]. Circadian genes are capable of influencing multiple levels of the TLR pathway. Indeed, CLOCK knockout mice demonstrate significantly decreased TLR9 expression, and PER2 knockout mice demonstrate changes in TLR9 expression, responsiveness, and adjuvant-induced adaptive immune responses in addition to decreased macrophage production of TNF-α and IL-12 [60]. Clinical studies corroborate the experimental evidence: Greater core circadian clock gene expression is associated with upregulated TLR-signaling pathway activation across multiple human tumor types [56]. In melanoma, BMAL1 overexpression is associated with TLR cascade and signaling pathways [66]. However, more work is needed to delineate directional influences of circadian disruption on TLR signaling in clinical samples and to determine whether circadian signals impact the ability of TLR9 agonists to effectively boost anti-PD-(L)1 immunotherapy.

Another immunosuppression pathway commonly used in immunotherapies involves the cytotoxic T-lymphocyte-associated antigen 4 (CTLA-4 or CD152) receptor. The action of CTLA-4 is in the lymph node, rather than in the tumor. CTLA-4 is expressed on activated CD8+ effector T cells but primarily affects two CD4+ T cell subsets: it downregulates T-helper cell activity and enhances T_REG_ immunosuppressive activity [62]. T_REGS_ constitutively express CTLA-4 to suppress immune function. Antibody blockade of CTLA-4 expression enhances T-helper cell-mediated immune responses and antitumor immunity. The CTLA-4 inhibitor, ipilimumab, has demonstrated some success in metastatic melanoma and ovarian cancers.

There is burgeoning evidencing that circadian parameters are capable of influencing CTLA-4 pathways. Higher expression of core circadian clock genes is related to T_REG_ expression among multiple cancers, though again the direction of this relationship varies by cancer type [56]. Higher BMAL1 expression is correlated with higher CTLA-4 expression in melanoma tumors and their infiltrating T lymphocytes [66]. Thus, the same implications observed for anti-PD-(L)1 therapy may hold true here; i.e., that clock gene expression may need to be taken into consideration when examining the efficacy of anti-CTLA-4 therapies.

In support, circadian signals hold promise as anti-CTLA-4 therapeutic complements given their role in an alternative immune pathway that induces T_H_17 differentiation and activation, thus boosting host anti-tumor immunity [77]. Because of the delicate balance in immune cell levels required to prevent autoimmunity, boosting T_H_17 production dampens T_REGS_ and, along with them, CTLA-4 levels to decrease immunosuppressive effects [78]. Direct circadian control of T_H_17 cell lineage development has been documented: BMAL2 controls T_H_17 development through the IL-21 pathway in mice [12]. BMAL1, CLOCK and REV-ERBα control the differentiation of T_H_17 cells by suppressing RORγt and by directly binding and repressing its promoter, NFIL3, in mice [79]. Pharmacologic supplementation with RORγ1 and RORγt agonists increases IL-17 production by T_H_17 human lymphocytes [80]. RORγ agonists, which enhance BMAL1 transcription, have also been shown to blunt T_REG_ cell activity; and a RORγt agonist has demonstrated promise for increasing the antitumor activity of T_H_17 cells after ex vivo chimeric antigen receptor (CAR)-T-cell expansion [33]. Intriguingly, pharmacologic melatonin has been shown to control NFIL3 signaling and prevent T_H_17 cell overproduction in mouse models of multiple sclerosis [50]. Taken together, these data reveal that circadian signals exert positive and negative feedback to control the immune cell balance, and this have direct implications for anti-tumor therapeutic strategies. BMAL1 expression again emerges as a promising candidate biomarker and complementary therapeutic target.

## Circadian hormones and immunotherapies

In addition to cell-cell interactions, circadian timekeepers use hormones to systemically regulate cell cycling. Circadian signals trigger the release of glucocorticoids to mobilize energy throughout the body. Peak endogenous expression typically occurs in the morning hours soon after awakening. Disrupted cortisol expression is strongly linked to tumor progression [81, 82] and survival in multiple cancer types [82–85]. Because glucocorticoids tightly control several aspects of immune function [2] and act as potent immunosuppressives, corticosteroid administration is the primary treatment modality for inflammatory and autoimmune processes. For the same reason, corticosteroids have the potential to influence immunotherapy efficacy.

While administration of corticosteroids in response to adverse events is generally not thought to impact anti-PD-(L)1 efficacy, both stress-induced and pharmacologic increases in circulating glucocorticoids that are present at the initiation of treatment appear to have deleterious effects on anti-PD-(L)1 response. In mice, chronic stress-induced rises in circulating glucocorticoids leads to a reduced ability to mount effective chemotherapy-induced and PD-1-blockade-induced anti-tumor immune responses; similar effects were observed after corticosteroid administration [67]. Importantly, parallel patterns were seen among a corollary sample of non-small cell lung and colorectal cancer patients when compared to healthy controls: higher distress was associated with elevated circulating glucocorticoids which were, in turn, associated with elevated expression of biomarkers indicative of suboptimal anti-PD-1 therapeutic response [67].

Retrospective clinical data similarly suggest that corticosteroids have deleterious effects on anti-PD-(L)1 response. Among melanoma patients with brain metastases treated with pembrolizumab, the use of 0.5–12 mg prednisone equivalents at the initiation of immunotherapy was associated with poorer progression-free and overall survival when compared to patients not receiving glucocorticoids [86]. In a similar study among non-small cell lung cancer patients, the use of ≥10 mg corticosteroids on the day of PD-(L)1 blockade initiation predicted reduced objective response rates and shorter progression-free and overall survival times; these findings were not explained by smoking history, performance status, or history of brain metastases [87]. While it is generally thought that patients receiving corticosteroids have poorer prognosis, the relatively balanced presentation at baseline of these patient groups suggests the possibility that corticosteroids have an independent, negative effect on immunotherapy.

Corticosteroid administration may also have deleterious effects on anti-CTLA-4 therapy outcomes. Endogenous glucocorticoids are capable of extensively modifying cytokine signaling and have been shown to inhibit IL-2 and IFNγ, both of which are implicated in resistance to anti-CTLA-4 therapy [88]. Similarly, dexamethasone is capable of suppressing effector T cells through the IL-2 pathway and can increase levels of T_REGS_ [87]. Among melanoma patients treated with high- versus low-dose corticosteroids for ipilimumab-induced hypophysitis, time to treatment failure and overall survival were both significantly shorter in patients treated with doses > 7.5 mg, while the two groups did not differ on any baseline clinical characteristics [88]. These findings highlight that starting corticosteroids after initiation of immunotherapy had a significant negative impact on clinical outcomes, contrary to general convention, and showed that patients who required corticosteroids were not experiencing poorer functional status at the start of the trial. Four separate retrospective analyses of ipilimumab clinical trial data compared patients receiving corticosteroids to patients who were not. Patients who were receiving corticosteroids at the time of immunotherapy initiation experienced poorer disease control and poorer overall survival [89, 90], whereas patients who were started on corticosteroids after the initiation of immunotherapy experienced some, but not significant, shortening of response duration or overall survival [91, 92]. Comparisons across trials are somewhat difficult as corticosteroid dosage amount, timing, and duration of use is not reported, nor is endogenous glucocorticoid expression status. Nonetheless, these studies demonstrate a potential negative effect of corticosteroid use on the efficacy of checkpoint inhibitors.

Circadian hormones are also important to consider in efficacy of complementary immunotherapy strategies. Oncolytic virus therapies represent a promising new form of immunotherapy adjuvant. Here, tumor tissue is compared to healthy tissue from the host to assess for neoantigens expressed in the tumor DNA. Peptides from these tumor-specific neoantigens are then synthesized and injected back into the tumor, which turns a “cold” tumor “hot,” and more capable of activating an immune response. Once a tumor becomes “hot,” immunotherapy is more effective. In a phase 1b clinical trial of a neoantigen vaccine for glioblastoma, it was shown that neoantigen vaccine was capable of initiating a robust T-cell response and lengthening survival, but only among patients who were not receiving dexamethasone at the time of vaccine administration [93]. Dexamethasone is commonly administered to glioblastoma patients to control brain swelling. Patients who were receiving even low-dose dexamethasone (< 8 mg/day) were not able to mount a CD8+ T-cell infiltrative response to tumor neoantigen and died sooner from their disease. Because these doses of dexamethasone are within physiological range, consideration of a patient’s endogenous glucocorticoid level and administration time-of-day for neoantigen vaccination could be of paramount importance for achieving an adequate response.

There are speculations about the mechanisms by which glucocorticoids impact immunotherapeutic responses. These may involve a reduction in the CD8+ T-cell proliferation needed for effective PD-(L)1 blockade [87], though glucocorticoids appear to affect proliferation of naïve CD8+ T cells more than activated CD8+ T cells [94]. Because many clinical trials exclude patients already on corticosteroids, sufficiently large retrospective analysis to answer remaining questions will be difficult. These intriguing findings warrant further, prospective study where the predictive capacity could potentially be increased through assessment of basal endogenous glucocorticoid diurnal fluctuations, close monitoring of corticosteroid dose and administration timing, along with comparison to a clinically and demographically similar, corticosteroid-free control cohort.

## Improving clinical measurement of circadian disruption

Emerging evidence strongly suggests that circadian signaling may play a role in the efficacy of immune-based anti-cancer therapeutics and may warrant pursuit in future clinical studies to improve our ability to predict which patients will respond favorably to immunotherapy. There are several important factors to consider when translating circadian science into the clinic. Perhaps most important is the time-of-day in circadian measurements. Because expression levels vary widely over the course of the day, associations with circadian-related health and disease processes may be missed if assessed at a time at which expression is suppressed. Similarly, discerning broader implications from studies represented in the literature is precluded if measurement time-of-day is not considered or reported. This poses an obvious challenge in clinical examinations, where the timing of tissue collection is often dictated by the timing and accessibility of clinical processes and procedures. Some variability could be overcome by limiting sample collection to only morning or afternoon hours and reporting the range of time in which samples were collected. By introducing this methodological control, studies in circadian misaligned organisms may more readily identify the nature and/or magnitude of misalignment within and across studies.

Predictive studies will become increasingly feasible with the continued development of wearable devices capable of assessing cyclic behavior patterns, such as objective ambulatory and body temperature monitoring [95]. Wrist-worn accelerometers offer reliable, continuous measurement of circadian systems and have been widely used to measure circadian rest/activity patterns and sleep parameters in humans [96, 97]. Circadian rest/activity periods derived from actigraphy demonstrate robust associations with quality of life impairments [98], and strong prognostic value for metastatic colorectal cancer [99, 100] and primary head and neck cancer patients [101]. Similarly, body temperature rhythms – a robust indicator of endogenous circadian function [102] – are directly driven by central circadian control of parasympathetic (vasodilation) and sympathetic (vasoconstriction) signals [103]. Body temperature monitoring can be performed noninvasively using wrist or thoracic sensors and has proven useful for optimizing the timing of cancer therapy [104, 105]. There have also been many recent advances in biomarker sampling that offer fast, relatively non-invasive methods for circadian timing detection using established or developing platforms. One such example is SkinPhaser, which assesses single epidermal samples and applies established algorithms (CYCLOPS, [106]; ZeitZeiger, [107]) to accurately estimate host circadian time [108]. New machine learning algorithms may hold promise for successfully predicting circadian clock dysfunction and patient prognosis from single tissue samples [109]. Importantly, in vitro data are revealing that assessing populations of cells, rather than individual cells per se, is a strategy that may prove more informative about the nature of circadian disruption on cellular proliferation [54]. There is also a growing repertoire of measures targeted at gaining insights into tumor behavior, including liquid biopsies and the increasing availability of comprehensive tumor genomic, epigenomic and proteomic profiling. Combining these rich sources of data would better permit clinicians to identify patients whose systemic physiology and/or tumors are displaying prototypical patterns of disruption that allow for early strategizing and potentially stratifying to more appropriate, effective and/or tolerable cancer therapeutic regimens. Personalizing the timing of therapy to align with idiosyncratic host cycles is becoming a clinically feasible approach that, compared to current chronotherapeutic strategies, may offer greater specificity and superior efficacy for cancer control.

Such considerations also have important implications for drug delivery. There is substantial cellular, animal and human evidence implicating circadian modulation of tumor response to radiation, chemotherapy and molecularly targeted treatments, as well as overall survival [110, 111]. These studies have spawned several lines of investigation into circadian-modulated therapies, including chronotherapeutic timing strategies [112]. Drug toxicity may be reduced, and drug efficacy enhanced, by optimizing delivery to the time of day when optimal pharmacokinetics may be achieved. This is likely due to several factors, including circadian control of enzymes and transporters involved in metabolism and excretion of drugs and biological molecules [113], as well as circadian/time-of-day variation in the pharmacologic susceptibility of malignant cells [22].

On the other hand, the circadian clock is also a promising druggable target. Pharmacologic manipulation of circadian signaling has the potential to reset rhythms in a circadian misaligned organism, which could facilitate appropriate timing of anti-cancer drug delivery to synchronize with times at which healthy cells would be most resistant to off-target effects. Several promising circadian-targeting compounds have also been developed that in-and-of themselves show strong anti-cancer effects in vitro and in vivo [35, 73, 74]. Not only are circadian agonists capable of inducing tumor cell death while producing no off-target (healthy cell) effects or toxicities, lethality has been observed in tumors depending upon upregulated signalers such as H-RAS, K-RAS, BRAF, PIK3CA, as well as in p53 null tumor cells [35]. Future clinical trials can combine these circadian-targeting strategies, administering therapies on personalized chronotherapeutic schedules while also delivering novel pharmaceutical agents that target the tumor’s runaway clocks. A combined and personalized approach such as this will likely be key to improving patient outcomes. Because circadian disruption can be attenuated by behavioral and pharmacological interventions, it is a promising target for extending life expectancy in patients with cancer.

## Tumor immunogenicity

With regard to biomarker identification, circadian gene expression changes appear to be more prevalent, and may be more appropriate as a biomarker, than gene mutation or copy number variation per se [56]. While individual circadian markers may prove useful for preemptively identifying individual tumor types that may respond to immunotherapy, combined indexing of core clock gene expression [56] offers an indication of the overall level of intratumoral circadian downregulation or reprogramming. While a circadian gene expression index was positively associated with better overall patient survival [56], it remains to be determined whether similar approaches can be used to predict response to immunotherapy. Use may also be limited by high cost and complexity of analysis. It may be more feasible to assess tumor-level expression of circadian proteins via immunohistochemistry, often performed as standard of care on solid tumors. With careful consideration for effects of time-of-day and other circadian-linked factors, this approach may allow for a more rapid clinical implementation of circadian assessments in combination with assessment of MMR deficiency, MSI, and other tumor characteristics. This would be informative for determining which tumors have retained, lost, or reprogrammed their circadian signals and how these tumors differ in their response to therapeutic strategies.

## Immune checkpoint inhibitors

There is also evidence supporting tight but complex associations between certain circadian biomarkers and immune checkpoint signaling in the tumor microenvironment. The most consistent evidence across multiple cancer types (lending greater clinical relevance) appears to be with BMAL1 and glucocorticoid expression levels. Intratumoral BMAL1 expression changes may signify tumor responsiveness to anti-PD-(L)1 immunotherapy [66]. Small molecules targeting circadian receptors that influence BMAL1 expression have demonstrated promise in early clinical trials as enhancers of anti-PD-(L)1 immunotherapy [74]. Limited pre-clinical data available suggests that BMAL1 expression may influence signals from CTLA-4 receptors [79] and TLR9 immune adjuvants [66]. In T_H_17 cells, BMAL1 and other circadian signals both positively and negatively control the balance between effector and regulatory T cells, which has direct implications for anti-tumor therapeutic strategies. However, whether BMAL1 can reliably serve as an indicator of anti-CTLA-4 therapeutic outcome, or influence TLR9 agonist efficacy either when used alone or in combination with anti-PD-(L)1 immunotherapy, are questions that remain unanswered.

Similarly, cancer patients who experience basal or pharmacologically induced glucocorticoid elevations appear to suffer suboptimal outcomes after anti-PD-(L)1 immunotherapy. The limited pre-clinical data available also suggest that glucocorticoid levels may influence signals from CTLA-4 receptors. The utility of evaluating basal glucocorticoid levels should be examined prospectively as a predictor of response to anti-PD-(L)1 and anti-CTLA-4 immunotherapy. Basal expression may also serve as an endogenous indicator of future likelihood for serious immune-related adverse events. Diurnal variations in endogenous cortisol expression may need to be factored into dosing considerations when systemic immunosuppression is warranted. Predictive studies of this nature – evaluating basal glucocorticoid levels prospectively as a predictor of response to immunotherapy – are important in consideration for future research. Mobile apps, such as the OnTimePoint Collection Management System (Salimetrics, Carlsbad, CA), have been designed to help overcome the challenges associated with accurate home-based collection of multi-point, noninvasive samples, and may facilitate prospective clinical work aimed at addressing these remaining questions.

Overall, it may be worth thinking about tumor-level circadian signaling in the same way we are beginning to think about therapeutic strategies, such as targeting MSI-high solid tumors, irrespective of site of origin, with a common therapeutic agent. There is some precedent for this; data shows similar patterns of circadian disruption predicts poorer overall patient survival across multiple cancer types (briefly reviewed in [33]). By comparing tumor circadian gene expression phenotypes with characteristics such as MMR deficiency, MSI, or PD-L1 expression, we may reveal an interaction that allows us to better classify patients according to their likelihood of adverse reaction or, more ideally, the likelihood of their tumor exhibiting an optimal response to immunotherapy.

## Conclusions

The exact nature of how circadian-controlled physiological processes interact at the host and tumor level remains poorly understood. What has become clear, however, is that focusing too closely on the cancer itself, and failing to consider the contributions of the host and their physiology, can be deleterious to cancer treatment advancements. Such a lack of insight stalls our potential to employ complementary/synergistic therapies that may improve patient outcomes where tumor-focused biological treatments have been stymied. Improving our understanding of these pathways will yield promising advances aimed at improving immunotherapy outcomes.

## Data Availability

N/A

## Abbreviations

Atg14: Autophagy Related 14
BMAL: Brain and muscle ARNT-like
CD: Cluster of differentiation
CDK: Cyclin dependent kinase
CDT: Chromatin licensing and DNA replication factor
CLOCK: Circadian locomotor output cycles kaput
CTLA-4: Cytotoxic T-lymphocyte associated protein 4
EGFR: Epidermal growth factor receptor
EMT: Epithelial mesenchymal transition
HDAC: Histone deacetylase
HLA: Human leukocyte antigen
IL: Interleukin
LINE-1: Long interspersed element-1
MAPK: Mitogen-activated protein kinase
MMP: Matrix metalloprotease
MMR: Mismatch repair
MSI: microsatellite instability
NAD+: Nicotinamide adenine dinucleotide
NFIL3: Nuclear factor, interleukin 3
PD-(L)1: Programmed death, programmed death ligand
PER: Period
PI3K: Phosphoinositide 3-kinase
Rb: Retinoblastoma
RORγ: Retinoic-acid receptor-related orphan receptor gamma
SCN: Suprachiasmatic nucleus
SIRT1: Sirtuin 1
T_H_: T-helper
TIM: Timeless
TLR: Toll like receptor
TMB: Tumor mutational burden
TNF: Tumor necrosis factor
T_REG_: T-regulatory
VEGF: Vascular endothelial growth factor
